# CD44v6 engages in colorectal cancer progression

**DOI:** 10.1038/s41419-018-1265-7

**Published:** 2019-01-10

**Authors:** Lixin Ma, Lihua Dong, Pengyu Chang

**Affiliations:** Department of Radiation Oncology, First Bethune Hospital of Jilin University, 130021 Changchun, China

## Abstract

CD44 is a transmembrane glycoprotein. When the *CD44* gene is expressed, its pre-messenger RNA (mRNA) can be alternatively spliced into mature mRNAs that encode several CD44 isoforms. The mRNA assembles with ten standard exons, and the sixth variant exon encodes CD44v6, which engages in a variety of biological processes, including cell growth, apoptosis, migration, and angiogenesis. Mechanistically, CD44v6 interacts with hyaluronic acid (HA) or osteopontin, or it acts as a coreceptor for various cytokines, such as epidermal growth factor, vascular endothelial growth factor, hepatocyte growth factor, and C-X-C motif chemokine 12. In this context, the receptor tyrosine kinase or G protein-coupled receptor-associated signaling pathways, including mitogen-activated protein kinase/extracellular-signal-regulated kinase and phosphoinositide-3-kinase/Akt, are activated. Using these actions, homeostasis or regeneration can be facilitated among normal tissues. However, overexpression of the mature mRNA encoding CD44v6 can induce cancer progression. For example, CD44v6 assists colorectal cancer stem cells in colonization, invasion, and metastasis. Overexpression of CD44v6 predicts poor prognosis in patients with colorectal cancer, as patients with a large number of CD44v6-positive cells in their tumors are generally diagnosed at late stages. Thus, the clinical significance of CD44v6 in colorectal cancer deserves consideration. Preclinical results have indicated satisfactory efficacies of anti-CD44 therapy among several cancers, including prostate cancer, pancreatic cancer, and gastric cancer. Moreover, clinical trials aiming to evaluate the pharmacokinetics, pharmacodynamics, efficacy, and toxicity of a commercialized anti-CD44 monoclonal antibody developed by Roche (RO5429083) have been conducted among patients with CD44-expressing malignant tumors, and a clinical trial focusing on the dose escalation of this antibody is ongoing. Thus, we are hopeful that anti-CD44 therapy will be applied in the treatment of colorectal cancer in the future.

## Facts

- Overexpression of CD44v6 predicts poor prognosis in colorectal cancer (CRC) patients.
- CD44v6 assists colorectal cancer stem cells in colonization, invasion, and metastasis.
- CD44v6 improves CRC resistance to anti-cancer therapy by stabilizing the cysteine-glutamate exchanger, increasing the expression of multidrug resistance genes, improving the formation of autophagosomes, and antagonizing the binding between Fas and Fas ligands.
- Current strategies of anti-CD44v6 therapy mainly include antagonizing the interaction between HA and CD44v6 and blocking the exon v6-encoded region by using a soluble peptide or the humanized monoclonal antibody.

## Open questions

- Due to the binding between the motif existing in the CD44 C terminus and the inhibitor of apoptosis-stimulating protein of p53 (iASPP), what is the impact of the iASPP-CD44 interaction on CRC progression?
- Because CRC stem cells highly express CD44v6, can this marker be used to reflect the burden of CRC stem cells in primary tumors, in metastatic lesions, or in circulating tumor cells?
- Because most CRC cases are characterized by Wnt activation and CD44v6 is a target of Wnt, is anti-CD44v6 therapy more selective for CRC patients with overexpressing CD44v6 tumors?

## Introduction

Colorectal cancer (CRC) significantly threatens public health. According to statistics from 2015, CRC has become the fifth leading cause of cancer-related deaths in China^1^. CRC is a heterogeneous disease because the molecular characteristics vary among cases. Accordingly, CRC can be subclassified into the microsatellite instability (MSI)-immune type, the canonical type, the metabolic type, and the mesenchymal type^2^. Profound alterations within these tumors include mutation in the *RAS* or *BRAF* genes, deficient mismatch repair (dMMR), somatic copy number alteration, and the CpG island methylator phenotype^2^. In addition, recent evidence suggests that CD44 plays an important role in cancer progression because it is capable of facilitating the colonization and metastasis of cancer stem cells^3^.

CD44 is a molecule located at the cell membrane^3,4^. The ectodomain, transmembrane domain, and cytoplasmic domain are composed of this molecule. The ectodomain of CD44 contains an N-terminal globular domain and a stem membrane-proximal region. The N-terminal globular domain provides the binding site for hyaluronic acid (HA)^4,5^. In humans, the gene encoding CD44 is located at the short arm of chromosome 11. The full-length *CD44* gene contains 20 exons and 19 introns^4^. The first five exons (1–5) and the last five exons (16–20) encode the N-terminal and C-terminal regions of the CD44 molecule, respectively^4^. Such exons are regarded as stable exons, which encode the CD44 standard isoform (termed “CD44s”)^4–6^. The intermediate exons (6–15) are referred to as variant exons^4^. By using the action of alternative splicing, variant exons are assembled with stable exons to form different messenger RNAs (mRNAs) encoding variant isoforms (termed “CD44v”)^4–6^. The specific role of a CD44 isoform is determined by the variant exon-encoded region^4,5^. Generally, three isoforms, including CD44s, CD44v6, and CD44v4-10, are detected in the human gut epithelium^7^. CD44v4-10 is highly expressed by intestinal stem cells^7^. Physically, this isoform assists in the development of the intestinal epithelium^7,8^. Under pathological conditions, this isoform drives adenoma formation^7,9^. Among familial adenomatous polyposis patients, the mRNAs encoding CD44v4-10 and CD44v6 exhibit significantly increased levels within adenomas compared to normal crypts, and CD44s expression is decreased^7^. Moreover, the hepatocyte growth factor (HGF)-mediated growth of intestinal organoids or adenomas in vitro depends on CD44v4-10 rather than CD44s^8^.

CD44v6 negatively impacts the prognosis of CRC patients^10–12^. As demonstrated, CRC cells with CD44v6 expression from patient-derived xenograft tumors generated metastatic lesions in recipient mice that differ from those in mice xenografted with CD44v6-negative CRC cells^13^. Hence, CD44v6 is engaged in CRC colonization, invasion, and metastasis. For example, CD44v6-positive CRC cells were detected as an increased nuclear accumulation of β-catenin, which initiated the transcription of genes associated with cell proliferation and epithelial–mesenchymal transition (EMT)^13^. Regarding EMT, it is engaged in the metastasis of epithelium-originated malignant tumors, and the relationship between CD44v6 and metastasis was first identified in pancreatic cancer, showing that if BSp73AS cells acquired CD44v4-7 (this isoform contains the exon v6-encoded region) or CD44v6-positive phenotype, these cells would have metastatic potential and form metastatic lesions in vivo^14^. Similarly, overexpressing CD44v6 among CRC cells increased their resistance to anti-cancer therapy. As previously reported, CD44v6-overexpressing SW480 cells exhibited superiority to conventional SW480 cells in resisting 5-fluorouracil (5-FU) or oxaliplatin by activating PI3K/Akt, mitogen-activated protein kinase/extracellular-signal-regulated kinase (MAPK/ERK), EMT, and autophagy-related signaling pathways^15^. In addition, CD44v6 stabilized the cysteine-glutamate exchange on the cell surface, thus protecting cancer cells against oxidative stress^16^. Reactive oxygen species (ROS) contributed to the killing effects of radiotherapy on cancer cells^17^. Collectively, CD44v6 is an obstacle in anti-cancer therapy.

In this review, we first compare the characteristics of CRC between the left and right sides of the colon. Then, the impact of CD44v6 on CRC prognosis and the relationships between CD44v6 and CRC locoregional invasion, CRC metastasis, and CRC gene mutation pattern are introduced according to recent advances. Next, we summarize the actions by which CD44v6 facilitates tumor growth, invasion, and metastasis during CRC relapse while elucidating the underlying mechanism of CD44v6-induced CRC resistance to anti-cancer treatment. Finally, the perspectives related to anti-CD44v6 therapy for CRC are analyzed according to the results from recent preclinical studies.

## CRC: “left” and “right”

CRC is a heterogeneous disease, not only because different degrees of cell differentiation exist but also because there are many cellular clones within the tumor^18^. At present, comprehensive treatment is the standard of care for these patients. However, the cellular subpopulations within the tumor exhibit different responses to the same type of anti-cancer therapy, thus causing the disease to be hardly treated^18,19^.

As extensively explored in recent years, the differences between the tumor occurring on the left colon and on the right colon have emerged in various aspects (Table 1). For example, the left colon discriminates its origin from the colon on the right side during embryonic development^20^. Additionally, patients with tumors on the right side of the colon commonly present systemic syndromes, including anemia, loss of body weight, or cachexia at the time of diagnosis, whereas patients with tumors on the left side of the colon always present with poor defecation, obstruction, or hemafecia. Moreover, the pathological features of tumors on different sides of the colon are varied. In addition to advanced T stage, the degrees of differentiation of tumors on the right side are often poor compared to tumors on the left side^21^. According to the consensus molecular subtypes of CRC, MSI-immune type and metabolic type are predominantly detected among patients with tumors on the right side of colon, while canonical type and mesenchymal type are commonly found among patients with tumors on the left side. Hence, the prognosis of CRC patients with canonical type is better than that of patients with any other subtype, whereas if patients experience CRC relapse, the risk of death in CRC patients with MSI-immune type is the highest among all cases^2^. Generally, the prognosis of late-stage CRC patients with primary tumors on the right side of colon is not as good as that of patients with tumors on the left side^22^.

**Table 1:** Summary of the characteristics of CRC on the left or right side

Characteristics	Left side	Right side	Author, ref.
Embryonic origin	Hindgut	Midgut	Minoo et al.^28^ Trosko et al.^20^
Blood supply	Inferior mesenteric artery	Superior mesenteric artery	Lee et al.^100^
Incidence at diagnosis*	57.1%*	42.9%	Meguid et al.^101^
Clinical presentation	Poor defecation, obstruction, or hemafecia	Anemia, body weight loss, or cachexia	Lee et al.^100^
Median size of primary tumor	40.0 mm	46.0 mm*	Meguid et al.^101^
T stage	T1–2 (7.0%) T3 (76.6%) T4 (16.4%)	T1–2 (4.5%) T3 (80.2%) T4 (15.3%)	Missiaglia et al.^102^
N stage	N0 (61.3%) N1 (26.3%) N2 (12.5%)	N0 (60.4%) N1 (24.3%) N2 (15.4%)*	Meguid et al.^101^
Degree of differentiation	G1 (12.3%) G2 (74.5%) G3–4 (13.5%)	G1 (9.5%) G2 (66.1%) G3–4 (24.5%)*	Meguid et al.^101^
Molecular subtypes	Canonical type and mesenchymal type	MSI-immune type and metabolic type	Guinney et al.^2^
Molecular characteristics	p53 (43.0%)* CD44v6 (51.2%) *BRAF*(3.4%) *KRAS* (36.6%) *PIK3CA* (11.0%) MSI-high (7.0%) Chromosomal instable (75%)	p53 (34.0%) CD44v6 (66.5%)* BRAF (15.7%)* KRAS (42.2%)^ns^ *PIK3CA* (20.0%)* MSI-high (30.1%)* Chromosomal instable (30%)	Soong et al.^103^ Minoo et al.^28^ Missiaglia et al.^102^ Shen et al.^104^
Predominant TIL at TAM	FoxP3^+^ Treg (rectum)	CD8^+^ CTL	Berntsson et al.^105^
Molecular pathways	MAPK/ERK Canonical Wnt	MAPK/ERK CpG island methylation-related serrated pathway	Missiaglia et al.^102^
Survival after radical surgery	5 years (59.7%)* 10 years (41.9%)* 15 years (29.5%)*	5 years (56.3%) 10 years (37.8%) 15 years (24.5%)	Meguid et al.^101^
Survival after first-line chemotherapy (Clinical Trial: NO16966 and CRYSTAL)	[FOLFOX4/XELOX]* **(NO16966)** Median PFS: 8.3 months; median OS: 22.0 months [FOLFIRI]* **(CRYSTAL)** Median PFS: 8.9 months; median OS: 21.7 months	[FOLFOX4/XELOX] **(NO16966)** Median PFS: 7.0 months; median OS: 17.0 months [FOLFIRI] **(CRYSTAL)** Median PFS: 7.1 months; median OS: 15.0 months	Loupakis et al.^106^ Tejpar et al.^107^
Efficacy of chemotherapy plus anti-EGFR therapy (Clinical Trial: CRYSTAL)	[FOLFIRI + cetuximab]* Median PFS: 12.0 months; median OS: 28.7 months	[FOLFIRI + cetuximab] Median PFS: 8.1 months; median OS: 18.5 months	Tejpar et al.^107^
Efficacy of chemotherapy plus anti-angiogenic therapy (Clinical Trial: AVF2017g)	[Chemotherapy + bevacizumab]* Median PFS: 11.1 months; median OS: 24.2 months	[Chemotherapy + bevacizumab] Median PFS: 8.7 months; median OS: 15.9 months	Loupakis et al.^106^
Immunotherapy	Anti-PD-1/PD-L1 only for dMMR/MSI	Anti-PD-1/PD-L1 only for dMMR/MSI	Yu^108^
Relapse pattern	Local (11.5%) Peritoneum (18.4%) Lung (19.2%) Liver (33.1%) Lymphatic node (12.3%) Other (5.4%)	Local (8.8%) Peritoneum (21.8%) Lung (16.9%) Liver (26.7%) Lymphatic node (16.9%) Other (8.8%)	Missiaglia et al.^102^
Median OS	89.0 months^a^	78.0 months	Meguid et al.^101^

* Representing significantly high, *p* ≤ 0.05; ns: representing no significance among groups, *p* > 0.05

*TIL* tumor-infiltrating lymphocytes, *TAM* tumor-associated microenvironment, *Treg* regulatory T cells, *CTL* cytotoxic T lymphocyte, *CpG* cytosine-phosphate-guanosine, *PFS* progression-free survival, *OS* overall survival, *PD-1* programmed death 1, *PD-L1* programmed death ligand-1

Bold values indicates registration number of the clinical trial

The CRC heterogeneity primes varied responses of tumor cells to anti-cancerous therapy. Hence, genetic, epigenetic, or microenvironment alterations are engaged in this process^23^. For example, a high frequency of *BRAF* mutation often confers tumors on the right side of the colon with primary resistance to anti-epidermal growth factor receptor (EGFR) therapy compared with tumors on the left side^22,24^. Moreover, after anti-EGFR therapy, wild-type CRC cells acquire mutations in* RAS, BRAF, PIK3CA, HER2, FGFR1, PDGFRA,*and *MAP2K1* solely or in combination^24,25^. Despite their low frequency, CRC cells with amplification of the *MET* gene also contribute to primary or secondary resistance to anti-EGFR therapy, as HGF can activate RTK signaling in parallel with EGF^8,24,26^. During this process, the intracellular portion of CD44v6 assists in linking the MET cytoplasmic domain to actin microfilaments and intermediating ezrin, radixin, and moesin proteins, thus facilitating the activation of RAS by son of sevenless^27^. Intriguingly, the percentage of CD44v6-positive cancer cells within tumors is higher in the right colon than in the left colon^28^. Moreover, recent evidence suggests that consecutive reprogramming of CRC stem cells highly express the mRNA encoding for CD44v6, implying the clinical significance of CD44v6 in CRC^13^.

## Clinical significance of CD44v6 in CRC

### CD44v6 and CRC prognosis

In the clinical setting, CRC patients with the same stage frequently show different outcomes even if receiving equivalent anti-cancer therapies^29^. Evidence suggesting that a high percentage of CD44v6-positive cells within tumors indicates poor survival has been revealed in several cancers, including gastric cancer, pancreatic cancer, osteosarcoma, lung cancer, esophageal cancer, hepatocellular cancer, and ovarian cancer^30–35^. Likewise, CD44v6 has emerged as an independent factor that inversely affects the survival of CRC patients^10–12^. Mechanically, CD44 gene expression is driven by canonical Wnt, which is unconventionally activated among 37% of all CRC cases^2,36^. Moreover, CRC stem cells with high expression of CD44v6 possess great invasive and metastatic potential^13^. Generally, CRC patients with or without CD44v6-positive cells in primary tumors display 5-year survival rates of 52.78% and 80.95%, respectively^37^.

### CD44v6 and CRC locoregional invasion

The invasiveness of CRC cells is determined by their degree of differentiation; cells that are poorly differentiated are more invasive than those that are moderately differentiated or well differentiated^38^. A previous study reported that the percentage of CD44v6-positive cells was closely related to the degree of CRC differentiation^37^. Within well-differentiated tumors, the percentage of CD44v6-positive cells was 18.18%. In contrast, this amount reached 67.65% in moderately differentiated tumors and 91.67% in poorly differentiated tumors^37^. Accordingly, the percentage of CD44v6-positive cells within primary lesions at Dukes A and B stages was 33.33%, whereas this percentage reached 84.85% at Dukes C and D stages^37^. Similar to this result, a previous study reported that if the primary lesion invaded into the muscle layer, subserosa, or extra subserosa at the time of diagnosis, then the CD44v6 expression was detected in 62%, 59%, or 82% of enrolled patients, respectively^10^. Additionally, CD44v6 expression at the primary lesion was only found in 66% of patients without regional lymph node involvement. In contrast, CD44v6 expression at the primary lesion was positively detected among 84% of patients possessing metastasis in one to three lymph nodes, and 86% of patients with metastasis in four or more lymph nodes possessed CD44v6 expression in their primary tumors^10^. Together, these results indicate that primary tumors bearing large amounts of CD44v6-positive cells are poorly differentiated, which enables the primary lesions to be at advanced T stages along with massive lymph node involvement at the time of CRC diagnosis.

### CD44v6 and CRC metastasis

Metastasis in patients with CRC represents a wide spectrum of diseases. Herein, the liver and lung are common organs affording CRC metastatic lesions. Among 20–25% of all CRC cases, metastatic lesions can be detected as soon as the primary lesion is diagnosed^39^. However, the prognosis of these patients is not always satisfactory. For example, there is a significant difference in survival when metastasis is detected at 1 month before diagnosis vs. more than 12 months after diagnosis, and the 5-year survival rates are 39% vs. 48%, respectively^39^. The results from a meta-analysis recently confirmed that CD44v6 overexpression was closely related to a high incidence of distant metastasis of CRC^40^. Consistently, this proposal is supported by the data from basic studies^13^. For example, CD44v6-positive CRC cells are apt to form metastatic lesions in the lung and the liver. Herein, the interaction between osteopontin (OPN) and CD44v6 has been revealed as a candidate in facilitating CRC liver metastasis^41^.

Nevertheless, CRC metastasis is triggered by multiple factors, not only by CD44v6. For example, CRC cells that simultaneously express CD44v6, FAK (focal adhesion kinase), EGFR, and MET are prone to metastasis^12^. Moreover, determining the percentage of circulating tumor cells (CTCs) in the peripheral blood of cancer patients to predict the burden of cancer metastasis has been applied. The percentage of CD133^+^CD44^+^CD54^+^ CTCs was positively correlated with the incidence of CRC liver metastasis^42^. At present, serum carcinoembryonic antigen testing and abdominal computed tomography or magnetic resonance imaging scans are recommended for monitoring CRC relapse during follow-up^29,43^. When combining CD133^+^CD44^+^CD54^+^ CTCs, the sensitivity and specificity in diagnosing CRC liver metastasis reach 88.2% and 92.4%, respectively^42^. Although CTC assessment is still not recommended in a clinical setting, the effect of CTC amount on the prognosis of CRC patients should not be ignored^21^.

### CD44v6 and CRC gene mutations

Due to the inconsistent molecular characteristics, drugs targeting molecules, including EGFR, VEGF (vascular endothelial growth factor), VEGFR, HER2, MEK, BRAF and MET, have been developed, and some of these have been approved for CRC treatment. However, one drug only kills cells sensitive to it^18,19^. Clones that resist this therapy will expand their numbers to provoke CRC relapse^18,19^. According to the current theory, there are mainly two cases involved in this process. In one case, clones possessing phenotypes primarily resistant to treatment triggered CRC relapse^18,19^. In the other case, when challenged by anti-cancer therapy repeatedly, a small portion of CRC stem cells evolved to possess adaptive phenotypes, which caused the treatment to be inefficient^18,19^. Irrespective of the case, it is critical that CRC stem cells acquire resistance to anti-cancer therapies by reprogramming their phenotype because CRC stem cells hierarchically produce all offspring possessing the resistant phenotype^18,19,23^. Reprogramming CRC stem cells are characterized by high expression of CD44v6^13,44^. Grillet et al.^44^ recently separated three CTC lines from chemotherapy-naive patients with metastatic CRC and compared the phenotypes among these CTC lines. CRC stem cell marker genes, including *ALDH1A1, CD133, EpCAM, CD44v6*, and* CD26*, were highly expressed by all CTC lines^44^. Nevertheless, the mRNA levels of CD44 were significantly greater in CTC lines than in primary and liver metastatic lesions^44^. In this context, the gene mutation patterns in some CTC lines were significantly different from those in their related primary lesions, mainly presenting *BRAF* mutations in CTC lines and* KRAS* mutations in primary lesions^44^. Herein, the primary resistance of CRC to anti-EGFR therapy is largely attributed to mutations of *RAS* and *BRAF*^21^, which are present in approximately 70% of all resistant cases^24,25^. Additionally, *BRAF* mutation testing is recommended if expression is missing for MLH1, a protein functioning in DNA MMR. Deficiency in MMR preferentially leads to MSI, and approximately half of patients with an MSI-immune phenotype present with poor differentiation and metastatic lesions at the time of CRC diagnosis^21,45^. As mentioned above, the frequency of the MSI-high phenotype is high in the right colon^2^. Accordingly, the frequency of *BRAF* mutation is positively associated with several characteristics of CRC, including the location of primary tumors on the right side of the colon and the poor differentiation of tumor cells^46^. Hence, CD44v6-positive CRC cells are reported to be frequently detected in tumors on the right side of the colon compared to the left side^28^. However, there are no significant differences in CD44 expression within primary lesions with the microsatellite-stable and MSI phenotypes^47^.

## Mechanisms underlying CRC progression due to CD44v6

Between 2010 and 2014, the 5-year survival rate of CRC patients in China was approximately 57%, which is significantly greater than that reported 10 years ago^48^. However, despite undergoing comprehensive treatment, CRC relapse is still unavoidable. It is estimated that more than 50% of CRC patients will progress and/or develop metastasis during their lifetime^49^.

### CD44v6 promotes CRC colonization

**Fig. 1:**
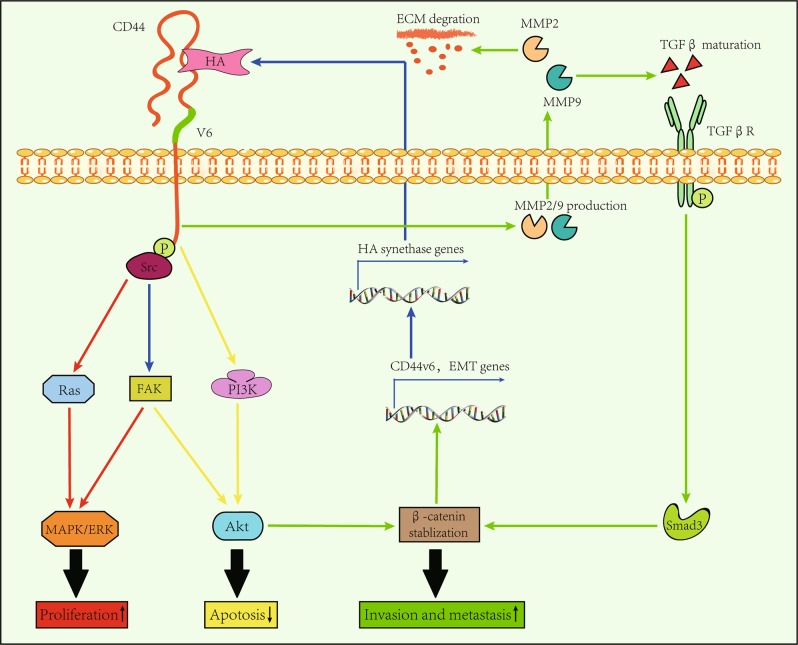
The interaction between CD44v6 and HA. HA interacts with the ectodomain of the CD44v6 molecule to facilitate CRC colonization, invasion, and metastasis. Furthermore, HA promotes the phosphorylation of the cytoplasmic domain of CD44v6, which then activates Ras and FAK through Src, thus resulting in activation of MAPK/ERK signaling pathway (red line). Likewise, the interaction between HA and CD44v6 also activates the PI3K/Akt signaling pathway, which increases the resistance of CRC cells to apoptosis (yellow line). Moreover, the interaction between HA and CD44v6 increases the production of MMP2/9, which degrades ECM along with promoting maturation of TGFβ (see ref. ^61^). After acting on its receptor, Smad3 will stabilize β-catenin intracellularly together with activated Akt. Then, the expression of EMT-related genes along with the gene encoding CD44v6 increase (see ref. ^71^). As a result, the invasive and metastatic capabilities of CRC cells are improved (green line). In addition, the CD44v6 supports the expression of the gene encoding HA synthases in a feedback manner, which further strengthens the above-mentioned effects

As mentioned above, the expression of CD44v6 increases upon malformation in the gut^7^. Carcinogenic conditions could alter post-transcriptional processes involving CD44 pre-mRNA alternative splicing, resulting in the existence of multiple CD44 isoforms within tumors^6,50^. Among these isoforms, CD44v6 exhibits superior affinity to HA compared to CD44s^51^. Functionally, HA is a critical extracellular matrix (ECM) component that promotes phosphorylation at the cytoplasmic domain of CD44, thus independently activating the MAPK/ERK and phosphoinositide-3-kinase/Akt (PI3K/Akt) signaling pathways^4,52^ (Fig. 1). CD44v6 supports the expression of the gene encoding HA synthase 3 in a feedback manner^53^. The ectodomain of CD44v6 can be decorated with chondroitin sulfate or heparin sulfate, which enables CD44v6 to bind to growth factors, such as EGF, VEGF, and HGF^52^. Unlike EGF and VEGF, the ectodomain of CD44v6 directly controls MET activation in an HGF-independent manner^52,54^. For example, previous studies have confirmed that in the presence of HGF, the phosphorylation of molecules including ERK, Akt, and MET is significantly decreased under CD44v6-absent conditions^55,56^. Collectively, CD44v6 directly potentiates RTK-associated signaling pathways and acts as a coreceptor for some growth factors, thus resulting in an improvement in cell proliferation and cell resistance to apoptosis and angiogenesis^57,58^.

### CD44v6 increases CRC invasion and metastasis

Invasiveness is a typical feature of cancer^59^. In addition, degradation of the ECM is critical for enlarging the space for tumor cell invasion^60^. The interaction between HA and CD44v6 modifies ECM components to support the invasiveness of cancer cells by stimulating the production of matrix metalloproteinase 2 (MMP2) and MMP9^61,62^ (Fig. 1). Furthermore, MMP2 potently degrades type IV collagen, which is the major component that forms the structure of the basement membrane^63^. When penetrating this barrier, CRC cells tend to invade into lymph nodes or distant organs^63^. MMP9 is also a candidate in promoting CRC metastasis^63^. For example, MMP9 enables the activation of tumor growth factor-β (TGFβ) by interacting with CD44v6^64^. TGFβ/Smad signaling induces EMT, which impacts CRC metastasis greatly^65^. Likewise, the EMT process can be triggered by Wnt^66,67^. Irrespective of their relationship, the Smad3 molecule prevents β-catenin degradation^68,69^. Thus, within the tumor, CD44v6-positive CRC cells at the invasive front possess obvious nuclear accumulation of β-catenin^67,70^. Within the nucleus, the interaction between β-catenin and Tcf-4 controls the transcriptional activation of genes associated with EMT and CD44v6^71^. By using RTK-associated signaling, HA-CD44v6 also supports the colonization of metastatic cells^4,52^.

**Fig. 2:**
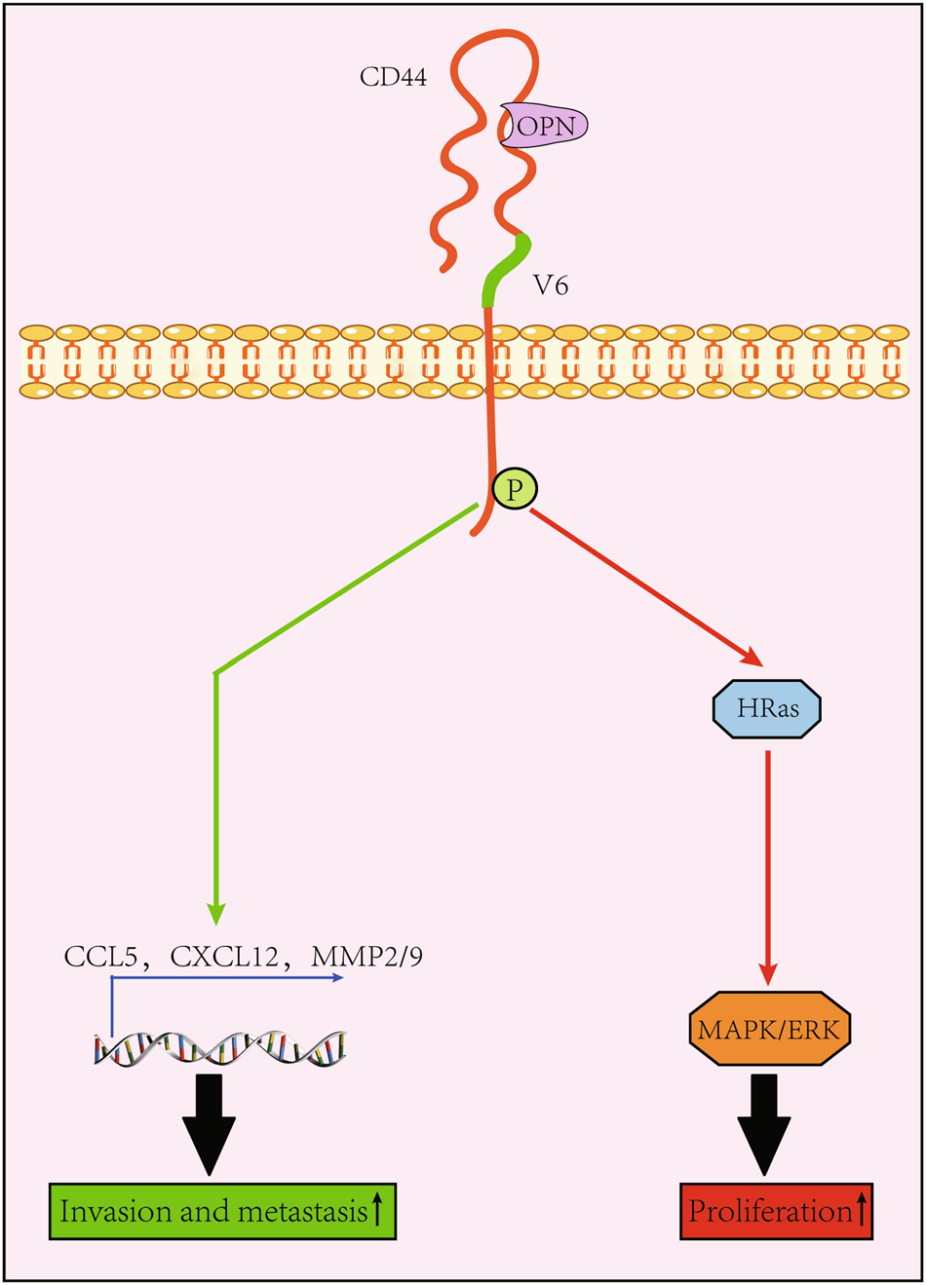
Interactions between CD44 and OPN. After binding to the ectodomain of the CD44v6 molecule, OPN is potent in inducing CRC colonization along with invasion and metastasis. Furthermore, OPN is able to activate the CD44v6 molecule through enabling the intracellular domain of the CD44v6 molecule to be phosphorylated, which is demonstrated to be able to upregulate the expression of genes encoding CCL5, CXCL12, and MMP2/9 (see ref. ^72^). As a result, the invasive and metastatic capabilities of CRC cells improve (green line). In addition, the interaction between OPN and CD44v6 is able to promote the proliferation of CRC cells by activating the HRas/MAPK/ERK signaling pathway (red line)

**Fig. 3:**
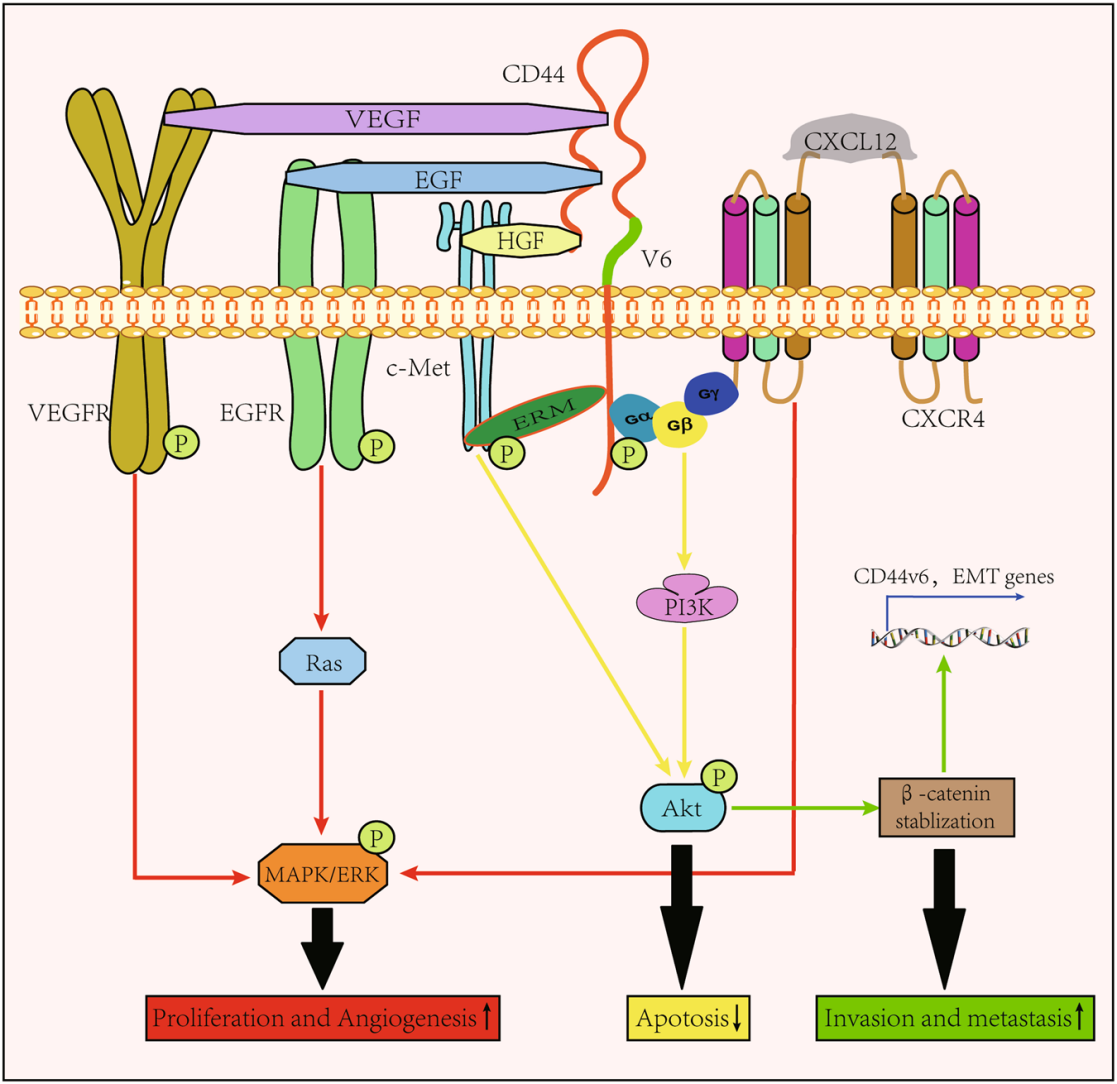
CD44v6 acts as a coreceptor for several cytokines. As a signal integration platform, the ectodomain of the CD44v6 molecule can be bound with several cytokines, such as EGF, HGF, VEGF, and CXCL12, which are known to be potent in inducing cell proliferation, anti-apoptosis, migration, and angiogenesis through activating MAPK/ERK signaling pathway (red line) and PI3K/Akt signaling pathway (yellow line) after binding with their receptors. Moreover, due to the existing crosstalk between PI3K/Akt and Wnt/β-catenin (see ref. ^58^), β-catenin translocates into the nucleus to promote the expression of EMT-associated genes, thus enabling the invasive and metastasis capabilities of CRC cells to be improved (green line)

In addition to HA, OPN is another molecule that interacts with CD44v6^4^. Functionally, OPN induces the distant metastasis of CD44v6-positive cells by increasing their chemotaxis^13,41,72^ (Fig. 2). Moreover, OPN assists metastatic cells in colonization by activating HRAS^73^ (Fig. 2). Similar to OPN, HGF and CXCL12 also mediate CRC metastasis. As previously demonstrated, when conditioned by HGF, CXCL12, or OPN, the CRC cells incompetent in metastasis acquire the CD44v6-positive phenotype^13^. After being xenografted into mice, these cells efficiently give rise to metastatic lesions compared to controls^13^. Concerning the function of CXCL12 in CRC progression, this cytokine impacts a variety of processes, including cell growth, survival, and migration, after interacting with CXCR4^74^ (Fig. 3). Blockage of the CXCL12-CXCR4 interaction reduces the CD44v6 expression by CRC stem cells^13^. Mechanically, in the presence of CXCL12, there is an interaction between intracellular portions of CD44v6 and CXCR4, whereas knockdown of CD44 expression impairs CXCL12-mediated CXCR4 signaling^4^. Collectively, these results suggest that CD44v6 is engaged in CRC metastasis.

### CD44v6 improves the resistance of CRC to anti-cancer therapy

Chemotherapy, radiotherapy, and molecule-targeted therapy help manage CRC patients at late stages. In general, the core effects of these therapies halt the proliferation among cancer cells along with subsequent induction of cancer cell death^75^. However, such effects are compromised if CD44v6 is expressed.

**Fig. 4:**
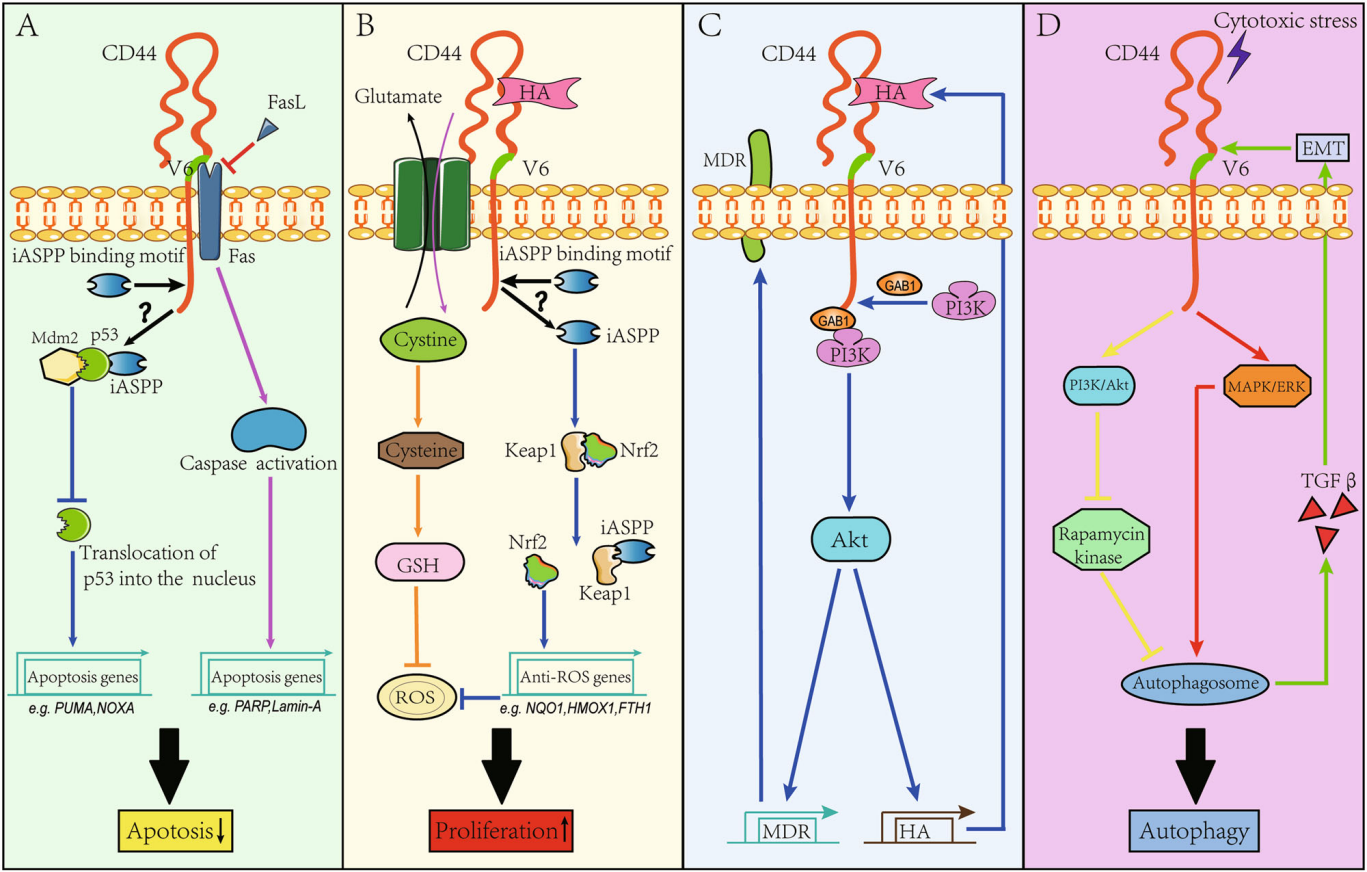
CD44v6 increases the resistance of CRC cells to anti-cancerous therapies. **a** CD44v6 is able to increase the resistance of CRC cells to apoptosis. Furthermore, iASPP binds to p53 together with Mdm2 to limit the translocation of p53 into the nucleus to initiate translocation of apoptosis-related genes, such as *NOXA* and* PUMA*. Hence, p53-dependent apoptosis is prohibited (see ref. ^77^) (blue line). Notably, although the C terminus of CD44v6 molecule contains a motif for iASPP binding, the biological effect of this binding remains unclear (denoted by the black question mark). In addition, CD44v6 maintains cell survival by competitively blocking the binding between Fas and their ligands (FasL), because the proximal membrane region encoded by the exon 6 variant provides a platform for Fas binding, which prevents caspase activation to limit cell apoptosis (see ref. ^96^) (pink line). **b** CD44v6 is able to promote proliferation among CRC cells. Thus, the interaction between HA and CD44v6 is able to stabilize the cysteine-glutamate exchanger on the cell membrane to increase the cytoplasmic level of cysteine, which then results in a high production of GSH. Therefore, GSH is able to suppress ROS (see ref. ^16^), protecting CRC cells against ROS-induced cell injury (orange line). In addition, iASPP clears intracellular ROS by binding with Keap1, thus enabling Nrf2 to be stabilized. Nrf2 will then translocate into the nucleus to initiate transcription of genes functioning in promoting cell expansion (see ref. ^78^) (blue line). **c** CD44v6 induces chemoresistance by increasing MDR gene expression and MDR activity. In this process, the HA-CD44v6 interaction is able to recruit the PI3K protein to the cytoplasmic domain of the CD44v6 molecule through the GAB1 protein (see ref. ^88^). By using this action, the PI3K/Akt signaling pathway is thereby activated. Furthermore, the genes encoding MDR and the enzyme engaging in the biosynthesis of HA are targets of the PI3K/Akt signaling pathway. MDR is known to pump intracellular toxins to the outside environment, thus protecting CRC cells against chemical agent-induced death. Moreover, by using the increased production of HA, the above process will be strengthened. **d** CD44v6 is able to induce chemoresistance by increasing autophagy. The activated CD44v6 will enhance the phosphorylation of both Akt and Erk under cytotoxic stress (see ref. ^15^). Furthermore, the activation of PI3K/Akt pathway suppresses the rapamycin kinase, which always acts as a negative regulator of autophagy activity (yellow line). Moreover, the activated MAPK/ERK signaling pathway also plays an important role in autophagosome induction (see ref. ^15^) (red line), which then acts on TGFβ/Smad signaling pathway to drive EMT (green line)

Chemotherapy- or radiotherapy-induced cytotoxicity involves p53^76^. However, the inhibitor of apoptosis-stimulating protein of p53 (iASPP) binds to p53 together with Mdm2 in a feedback manner, thus preventing the translocation of p53 into the nucleus to initiate the transcription of genes, such as *PUMA* and *NOXA*, that are associated with apoptosis^77^ (Fig. 4a). Independent of p53, iASPP clears intracellular ROS by competing with Nrf2 for Keap1 binding and then stabilizes Nrf2, which translocates into the nucleus to drive the transcription of genes associated with cancer growth and 5-FU resistance^78^ (Fig. 4b). RelA is another transcription factor that interacts with iASPP within the nucleus^79,80^. Functionally, RelA is anti-apoptotic^81^. A previous study found that iASPP cleaved by caspase proteins potentiated the inhibition of the transcriptional activity of RelA under cytotoxic stress^80^. There is a structural binding motif for iASPP in the C terminus of the CD44 molecule^79^. However, the impact of the iASPP-CD44 interaction on the transcriptional activities of p53, Nrf2, and RelA remains unclear. Nevertheless, a relationship between p53 and CD44 has been indicated, suggesting that p53 counteracts CD44-mediated proliferation and anti-apoptosis in addition to inhibiting CD44 expression^82^. Moreover, knockdown of *CD44v6* expression significantly increased the chemosensitivity and radiosensitivity of prostate cancer cells in vitro^83^. However, the *p53* gene is generally mutated or depleted among CRC cells, thus enabling the antagonized effects of p53 on CD44v6 expression to be abolished^84^. In this context, CRC cells increase their resistance to apoptosis^85^. The multidrug resistance (MDR) gene also participates in chemoresistance among CRC cells^86^. The MDR gene encodes P-glycoprotein, which is widely distributed among the intestinal epithelium and functions by pumping intracellular toxins into the lumen^87^. However, the HA-CD44v6 interaction increases MDR expression by activating the PI3K/Akt- or ErbB2-related RTK signaling pathway^88^ (Fig. 4c). Moreover, the activity of P-glycoprotein is increased upon PI3K/Akt activation, thus increasing chemotherapy failure among CRCs^89^. In addition to CD44v6-induced apoptosis evasion, autophagy is an important self-protective action conferring CRC cells with increased resistance to chemotherapy^90^. In this process, CRC cells consume energy from damaged materials within autophagosomes to maintain their survival^90^. Concerning the relationship between CD44v6 and autophagy, a recent study revealed that overexpression of CD44v6 serves as an inducer for the intracellular formation of autophagosomes^15^. Interestingly, increased autophagy enables cancer cells to upregulate CD44 expression^91,92^ (Fig. 4d). Based on these actions, resistance to chemotherapy is well maintained. Taken together, the results of these studies suggest that CD44v6 contributes to chemoresistance by inducing apoptosis evasion and/or autophagy. Thus, massive autophagy occurrence by tumor cells predicts poor prognosis in CRC patients^90^.

Anti-EGFR is currently recommended for treating late-stage CRCs without *RAS* and *BRAF* mutations, and anti-angiogenesis therapy is suitable for patients with *RAS* or *BRAF* mutation^21^. Despite its low incidence, *MET* amplification is involved in CRC relapse after multiple anti-EGFR therapies, as HGF activates RTK-associated signaling pathways independent of EGF in mediating CRC growth^8,24,26^. The specific effect of CD44v6 on MET activation is mentioned above^55,56^. CD44v6 acts as a coreceptor for VEGF, which decreases the effects of anti-angiogenesis therapy. Moreover, the recent appearance of immune checkpoint inhibitors, including CTLA-4, PD-1, or the PD-L1 mAb, has indicated that CRC patients with the MSI-high phenotype may benefit from this therapy because of the infiltration of large amounts of CD8^+^ cytotoxic T lymphocytes (CTLs) within tumors^24,93,94^. CTLs induce cancer cell death in a Fas/Fas ligand-dependent manner^95^. However, the exon v6-encoded extracellular region of the CD44 molecule serves as a fundamental binding site for Fas, thus preventing the interaction between Fas and Fas ligands^96^ (Fig. 4a).

## Anti-CD44v6 therapy in CRC

**Fig. 5:**
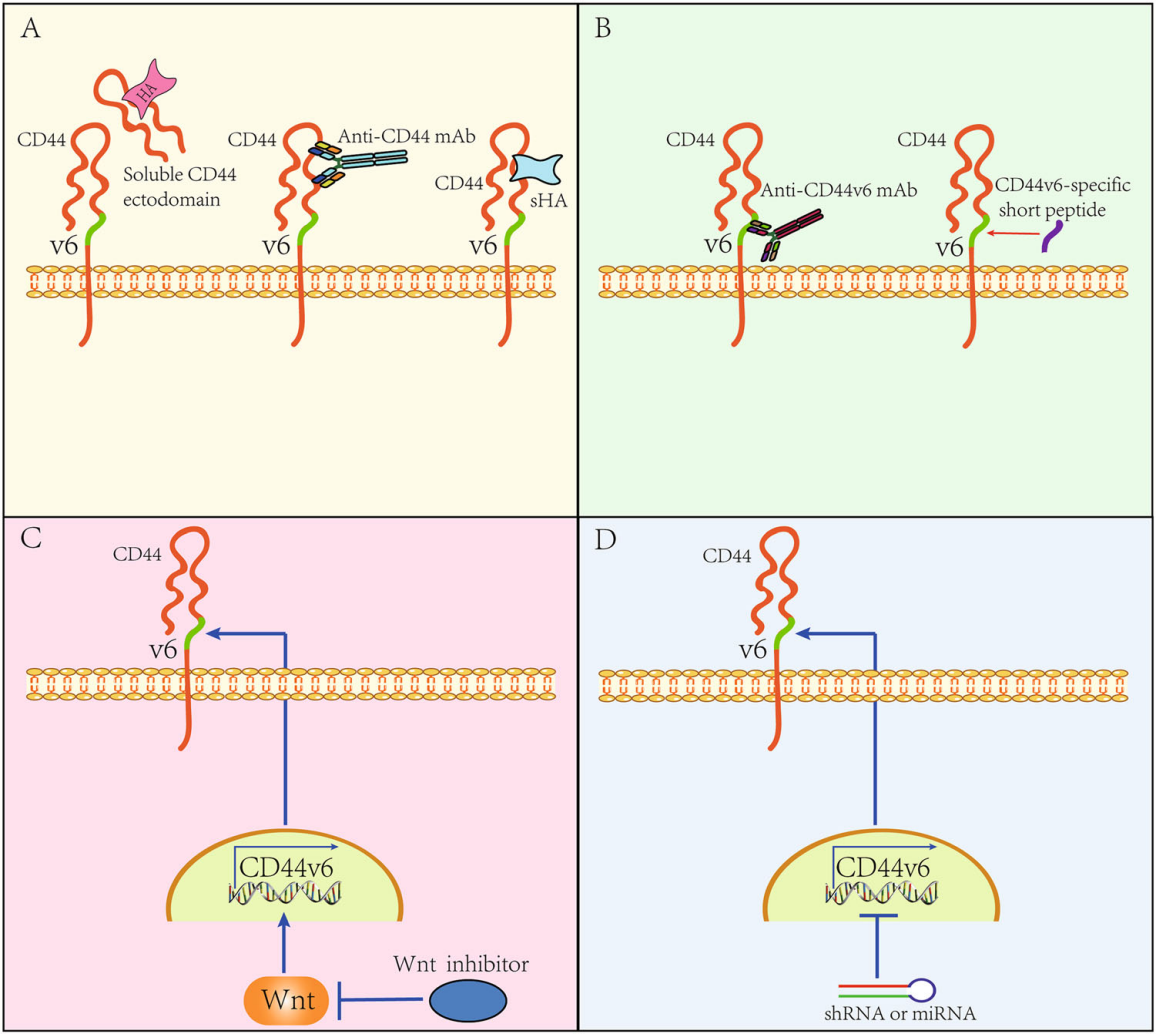
The strategies of anti-CD44v6 therapy. **a** The strategy of anti-CD44v6 therapy by blocking the interaction between HA and CD44v6. Furthermore, the soluble CD44v6 ectodomain is used for blocking HA binding, and the anti-CD44 mAb is used for blocking the HA-binding epitope on the CD44v6 ectodomain. In addition, the small fragment of HA (sHA) is able to inhibit the binding of HA to the CD44v6 ectodomain as well. **b** The strategy of anti-CD44v6 therapy by blocking the proximal membrane region encoded by the variant exon 6. Thus, the anti-CD44v6 mAb and CD44v6-specific peptide are available. **c** The strategy of anti-CD44v6 therapy by antagonizing the Wnt signaling pathway. Furthermore, the CD44v6 expression is driven by the activation of canonical Wnt (see ref. ^36^). Selective inhibition of Wnt by using small-molecule compounds potently suppresses the CD44v6 production (see ref. ^71^). **d** The strategy of anti-CD44v6 therapy by using shRNA or miRNA. Either miRNA or shRNA can effectively limit the CD44 gene expression, thus reducing the production of CD44v6 (see ref. ^3^^,^^5^)

Upon exploring the role of CD44v6 in CRC progression, several anti-CD44v6 strategies have been developed; several of these strategies aim to antagonize the interaction between HA and CD44v6 by using the soluble CD44 ectodomain to block HA binding, by using the α-CD44-HABD mAb to block the HA-binding epitope on the CD44 ectodomain, or by using the small fragment of HA (sHA), which inhibits the binding of HA to the CD44 ectodomain (Fig. 5a). Other strategies mainly target the exon v6-encoded region by developing an α-CD44v6 mAb or by synthesizing a CD44v6-specific peptide^3,5^ (Fig. 5b). In preclinical studies, these strategies have been confirmed to be effective in limiting cancer progression to varying degrees (for details see refs. ^3,]^^5^). The α-CD44v6 mAb and the CD44v6-specific peptide are promising for CRC treatment because they effectively inhibit MET and VEGFR2 signaling^5^. According to a recently published case, CRC patients with *MET* amplification and *BRAF* mutation could benefit from an ALK-MET inhibitor (crizotinib) plus a BRAF inhibitor (vemurafenib)^97^. In this respect, it is reasonable to speculate that drugs targeting CD44v6 will minimize the effects of *MET* amplification on CRC progression in combination with crizotinib, as CD44v6 activates MET signaling independent of HGF^55,56^. The results reported by Matzke-Ogi et al.^98^ support this proposal. Thus, the CD44v6-specific peptide is more effective at sensitizing human pancreatic cancer cells to apoptosis than crizotinib and the VEGFR2 inhibitor pazopanib, thus preventing tumor growth and metastasis more efficiently^98^. Because this peptide acts as a coreceptor for various growth factors, future works should focus on determining the efficacy of anti-CD44v6 therapy in treating CRC patients. Moreover, strategies targeting CD44v6 gene expression may be promising in a clinical setting, as results from basic studies have confirmed that the pharmacological inhibition of Wnt potently treats CRC^71,99^ (Fig. 5c). Alternatively, targeted inhibition of CD44 expression is also available by using shRNA (short hairpin RNA) or miRNA^3,5^ (microRNA) (Fig. 5d).

## Conclusion

CD44v6 significantly affects a variety of processes involving CRC progression, implicating CD44v6 as a candidate target for the treatment of CRC.
